# Cell encapsulation in liquified compartments: Protocol optimization and challenges

**DOI:** 10.1371/journal.pone.0218045

**Published:** 2019-06-21

**Authors:** Clara R. Correia, Maryam Ghasemzadeh-Hasankolaei, João F. Mano

**Affiliations:** CICECO–Aveiro Institute of Materials, Department of Chemistry, University of Aveiro, Aveiro, Portugal; University of Massachusetts Boston, UNITED STATES

## Abstract

Cell encapsulation is a widely used technique in the field of Tissue Engineering and Regenerative Medicine (TERM). However, for the particular case of liquefied compartmentalised systems, only a limited number of studies have been reported in the literature. We have been exploring a unique cell encapsulation system composed by liquefied and multilayered capsules. This system transfigured the concept of 3D scaffolds for TERM, and was already successfully applied for bone and cartilage regeneration. Due to a number of appealing features, we envisage that it can be applied in many other fields, including in advanced therapies or as disease models for drug discovery. In this review, we intend to highlight the advantages of this new system, while discussing the methodology, and sharing the protocol optimization and results. The different liquefied systems for cell encapsulation reported in the literature will be also discussed, considering the different encapsulation matrixes as core templates, the types of membranes, and the core liquefaction treatments.

## Introduction

Cell encapsulation is a very promising method for the treatment of different endocrine diseases, including diabetes, liver and kidney failure. There are also advantages in using this methodology in Tissue Engineering and Regenerative Medicine (TERM), including mediation of undesired acute immune responses, while offering a three-dimensional (3D) environment essential for anchorage dependent cells [1]. In the particular case of liquefied cell encapsulation systems, a possible strategy to build the encapsulation membrane is through the Layer-by-Layer (LbL) technique, which has been proposed to process materials for a variety of biomedical applications [2]. It is a simple, low cost, and cell-friendly method which can be performed under mild conditions using different pairs-interactions [3]. Moreover, LbL is a very versatile technique which allows us to produce membranes with desired permeability according to the number and composition of the layers deposited[4], while allowing to increase the applicability of the system by endowing nanoparticles [5] or lipids and viruses [6] into the multilayers.

The most important issue for liquefied cell encapsulation systems is the fact that most of the cells are anchorage dependent, so they need a solid surface to perform basic cellular processes. To overcome this problem, Correia *et al*. introduced a system in which microparticles are co-encapsulated with cells and used as a substrate [7]. In this system microparticles can possess a dual functionality *(i)* by offering physical support for cells, and also *(ii)* by controlling the differentiation fate of the encapsulated stem cells. For that, the surface of the microparticles was functionalized with plasma treatment to enhance cell adhesion affinity, and then subsequently coated with collagen I [8–10] or collagen II/TGF-β3 [5] in order to trigger osteogenic or chondrogenic differentiation, respectively. Others, have also used microparticles to encapsulate cells in a semi-liquid system, and showed their positive effect on cell viability [11].

Among the multiple methods for cell encapsulation, microfluidic techniques [12] and electrohydrodynamic atomization (EHDA) are the most common techniques to produce liquefied systems. Microfluidic techniques have a number of appealing features, since it allows a high control over the diameter and morphology of the cell encapsulation matrix. However, it requires a high gelation capacity of the encapsulation matrix solution, which limits the list of available biomaterials [13, 14]. Moreover, oil is often used as a continuous phase to aid forming droplets, which is not a suitable choice for biomedical applications [12]. On the other hand, EHDA has been increasingly used for high throughput production of particles in micro/nano scale with uniform size and morphology. In this method, a voltage is applied to cut the droplets from the needle tip before they fall as a result of gravity [15]. This technique is of great interest due to its easiness to operate, versatility, cytocompatibility, high efficiency, and the ability to work in a sterile environment [15–17].

**Fig 1:**
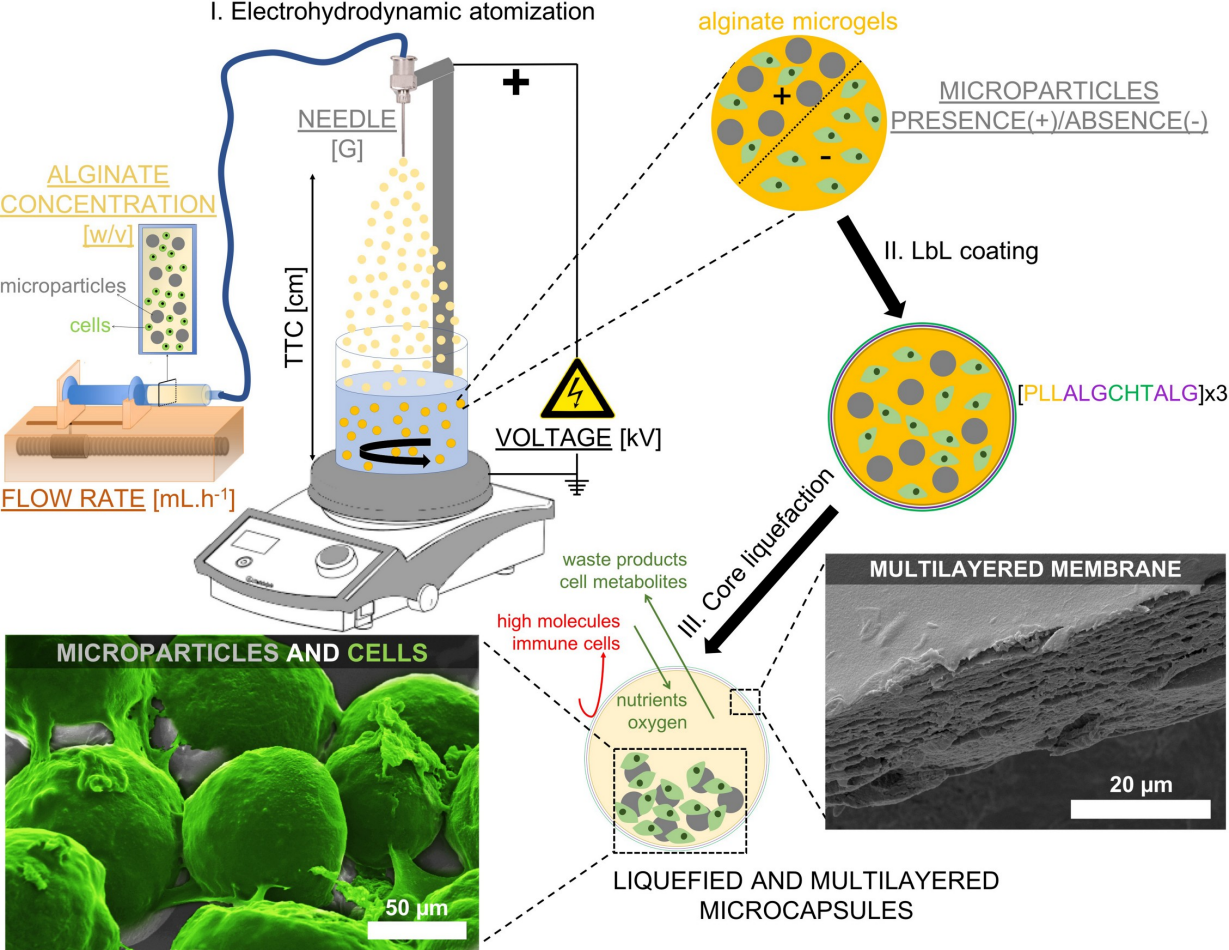
Schematic representation of the production of liquefied and multilayered microcapsules for tissue regeneration. (I) An alginate solution containing the cargo components, namely cells and microparticles, is electrosprayed by the electrohydrodynamic atomization (EHDA) process. The influence on the microgels diameter of different parameters of the EHDA process is tested, namely the voltage (kV), the flow rate (mL.h^-1^), the needle diameter (G), the distance between the tip of the needle to the collector (TTC, cm), the presence (+) or absence (-) of microparticles, and the alginate concentration (w/v). The different parameters tested are underlined. (II) Microgels are then used as templates to produce a multilayered membrane by Layer-by-Layer (LbL) technique using poly(L-lysine) (PLL), chitosan (CHT), and alginate (ALG) polyelectrolytes (n = 12-layers). (III) Ultimately, microgels are immersed in ethylenediaminetetraacetic acid, and liquefied and multilayered microcapsules are obtained. The LbL membrane of microcapsules is permeable to essential molecules for cell survival, while avoiding the entrance of high molecules and immune cells. On the other hand, the microparticles dispersed within the liquefied environment provide adhesion sites required for multiple cellular processes. Scanning electron microscopy images show a LbL membrane (adapted from reference [7]), and L929 cells (colored in green) adhered to the surface of microparticles (adapted from reference [8]).

In this paper, we describe how to apply the EHDA technique to prepare liquefied and multilayered microcapsules, which constitutes a clear important advance with respect to our pioneering cell encapsulation protocol proposed before [5, 7–10]. This allowed, for the first time, to prepare microsized capsules with controlled size. Briefly, the system is composed by a solid-core matrix encapsulating cells and solid microparticles. The solid-core matrix acts as a template enabling the build-up of a multilayered membrane by the LbL technique. Afterwards, core liquefaction is achieved using ethylenediaminetetraacetic acid (EDTA) as a chelator, removing the calcium ions from the egg-box structure of the crosslinked alginate. The encapsulated microparticles and cells can freely move inside the liquefied core, which gives cells freedom to self-organize into three-dimensional (3D) structures. While all the necessary cargo is wrapped into a single 3D structure, the permeable LbL membrane allows the diffusion of essential molecules for cell survival, such as oxygen, nutrients, metabolites, and waste products. On the other hand, microparticles offer a solid surface for the adhesion of the encapsulated anchorage-dependent cells, enabling different cellular processes (e.g., proliferation or differentiation). In particular, in this review we will discuss how to control the size of alginate microgels, which will serve as core templates for the production of liquefied and multilayered microcapsules. For that, different parameters of the EHDA process will be tested, namely, the voltage, the flow rate, the needle diameter, the distance between the needle and the collector (TTC), the presence/absence of microparticles, and the alginate concentration (Fig 1). We will also discuss other liquefied cell encapsulation systems reported in the literature.

## Liquefied systems for cell encapsulation

### Encapsulation matrix as core template

Cell encapsulation systems can be divided in two categories when considering the state of the core, namely into solid-core or liquid-core systems. It has been reported that there are some biological limitations in the solid-core matrix systems, namely a reduced cell growth and protein production, long-term stability, and also low rate of nutrient exchange, especially at the inner regions [18]. On the other hand, liquid-core matrix has better diffusion of essential factors required for cellular processes, which leads to higher cell viability and proliferation [8]. Sun *et al*. [19] showed the importance of the microenvironment on the behavior of encapsulated cells. Results show that a liquid-core matrix presented an increasing intracellular glycerol content and stress-tolerance substances in comparison with a solid-core matrix. Furthermore, cells extracted from the liquid-core matrix showed more resistance to hyperosmotic stress, oxidative stress, and heat shock stress than cells released from solid-core matrix. Kim *et al*. showed the importance of liquid-core cell encapsulation systems by comparing the viability of NIH 3T3 fibroblast cells in liquid and semi-liquid cores. Results showed that the encapsulated cells exhibited higher viability and growth in the liquid-core system [11].

Matrixes for cell encapsulation should allow mild gelation conditions. Alginate is the most common polymer used in bioencapsulation systems due to a number of appealing features. For the particular case of liquid-core/shell systems, almost all of the systems reported in the literature use alginate as the encapsulation matrix crosslinked through ionotropic gelation with calcium. An interesting exception reported in the literature is the work of Cellesi *et al*. [20], in which a fully synthetic substitute of alginate is proposed to produce liquefied spherical cell encapsulation systems, namely with a dextran core crosslinked using thermal gelation with Pluronic F127. Besides alginate, Sakai et al. [21] used unmodified gelatin to produce liquefied systems for the encapsulation of rat adipose-derived stem cells.

### Membrane composition

Due to its biocompatibility and versatility, LbL technique has been often used for the build-up of the membranes that surround the liquid core of cell encapsulation systems. The sequence of the polyelectrolyte combination and the number of layers can be varied according to the purpose and also the size of the capsules, since larger capsules require a higher number of layers for a proper core stabilization. For example, Costa *et al*. encapsulated osteoblast-like cells in 8 layers of chitosan/alginate [18]. Zhang *et al*. also encapsulated mesenchymal stem cells within 2 layers of chitosan/alginate to obtain liquefied capsules [22]. Correia *et al*. used a triple polyelectrolyte system composed by poly(L-lysine)/chitosan/alginate to have a 12-layered membrane [8, 10]. Additionally, in order to confer magnetic-response ability to liquefied capsules, Correia *et al*. incorporated magnetic nanoparticles within the multilayers, evidencing the versatile feature of the LbL technique [5]. The polyelectrolyte complexation of alginate (core) and poly(L-lysine) (shell), as pioneered by Lim and Sun [23], was also reported to produce liquid-core/shell fibers as cell encapsulation systems [24,25]. Besides alginate/chitosan as cell encapsulation membranes, modified gelatin incorporating phenolic hydroxyl groups was used [21]. The resultant membrane of crosslinked gelatin via a horseradish peroxidase catalysed reaction presented high cell adhesiveness and proteolytic biodegradability. Importantly, the gelatin membrane maintained its stability after incubation at 37°C. Synthetic polymers were also reported as membranes for cell encapsulation. Wu *et al*. also developed a core-shell microfiber system for the encapsulation of pheochromocytoma 12 cells [26]. The microfibers were prepared by coextrusion electrospinning method, using polycaprolactone (PCL) as the sheath and polyvinyl alcohol (PVA) as the core.

### Core liquefaction treatments

Alginate crosslinked by calcium ionotropic gelation is the most common method to produce cell encapsulation systems. To reverse this physical crosslinking one can use calcium chelators, namely ethylenediaminetetraacetic acid (EDTA) [5, 7–9, 18, 27] or sodium citrate [24, 28]. For example, Sugiura *et al*. [28] liquefied calcium alginate gel micro-fibers coated by poly(L-lysine) with 55 mM sodium citrate. Correia *et al*. [5, 7, 8, 10, 29] reported the use of 0.02 M EDTA for 5 minutes at room temperature to liquefy alginate cores. Another strategy is the use of temperature to liquefy gelatin cores, as demonstrated by Sakai *et al*. [21]. In this method, the liquefaction of the core was performed by incubation of the system at 37°C.

## Multilayered and liquefied microcapsules for tissue regeneration

### Methodology principles

Liquefied and multilayered capsules are composed by three essential elements, namely **(1) surface functionalized microparticles**, which provide adhesion sites for cells within the liquefied environment; they also can act as bioinstructive materials, for example to aid stem cells differentiation into specific lineages, such as into chondrogenesis [5] or osteogenesis [9, 10]; **(2) cells**, which are freely dispersed within the liquid core microenvironment and can self-organize into a 3D culture according to their specific needs. Different cells can be used and combined in multiphenotypic co-cultures, such adipose-derived stem cells alone [5] or co-cultured with adipose-derived microvascular endothelial cells [9, 10]; and **(3) a permselective multilayered membrane** that wraps the liquefied core of the capsules, ensuring permeability to essential molecules for cell survival while avoiding the entrance of high molecules and cells from the immunological system, although it is expected a minimal and controlled immune reaction due to the biotolerability of the materials employed. Additionally, the membrane confers flexibility to the capsule, which can aid to maintain its integrity during implantation by injection, and maximizes the direct contact between the core contents, namely cell-cell and cell-microparticle interactions.

### Protocols

#### Microparticles preparation

Polycaprolactone (PCL, Mw 80,000, Sigma-Aldrich) microparticles are produced by oil/water (o/w) emulsion technique. A 5% w/v of PCL/dichloromethane solution is prepared by dissolving PCL (1g) in dichloromethane (20 mL). This solution is slowly added to a 0.5% w/v aqueous (100 mL distilled water) polyvinyl alcohol solution (PVA, Mw ~ 30,000–70,000, Sigma-Aldrich) with the aid of a 21 Gauge (G) needle. The resulting dispersion is stirred at 500 rpm for two days to evaporate the organic solvent at room temperature (RT). Then, microparticles are washed in distilled water several times, until PVA removing. Microparticles are collected by centrifugation (500g, 5 min). Using this method, microparticles present a diameter range of approximately 45–100 μm (Dv10 = 46.4 μm, Dv50 = 70.1 μm and Dv90 = 104 μm, Mastersizer 3000, Malvern). If narrow diameter ranges are required, microparticles can be easily separated by sieving. For example, to narrow the diameter range of 45–100 μm to 50–60 μm, sieve the microparticles combining diameter meshes of 50 and 60 μm, and collect the microparticles in between. Ultimately, after washing with ethanol, microparticles are dried in the oven, overnight (**see Note 1**).

#### Microparticles surface modification with plasma treatment

Plasma treatment technique is used to modify the surface of microparticles. PCL microparticles are placed inside a reactor chamber fitted with a radio frequency generator at RT. Air is used as the working atmosphere. When the pressure of chamber is stabilized to ~0.2 mbar, a glow discharge plasma is created by controlling the electrical power at 30 V for 15 min (**see Note 2**).

#### Microparticles protein surface modification

To ensure cell adhesion, a coating material is employed at the surface of microparticles. Different coating materials known to enhance cell adhesion can be employed, such as collagen I [9,10] or collagen II [5] when aiming bone or cartilage tissue engineering applications, respectively. The amount of material employed to coat a certain amount of microparticles is calculated in mass per cm^2^. For the surface area calculation, it is considered the value of the Dv50 for the diameter. Then, PCL microparticles are sterilized by immersion in 70% v/v ethanol for 2 h. After washing with sterile phosphate buffered saline solution (PBS), microparticles are immersed in the calculated amount of coating material. After washing with PBS, microparticles are collected by centrifugation (500 g, 5 min) and stored at 4°C until use.

#### Alginate microgels production

The core materials, namely microparticles and cells, are loaded into an alginate solution that is subsequently extruded by EHDA to obtain microsized hydrogels. These alginate microgels will serve as template for the multilayered membrane production.

**Fig 2:**
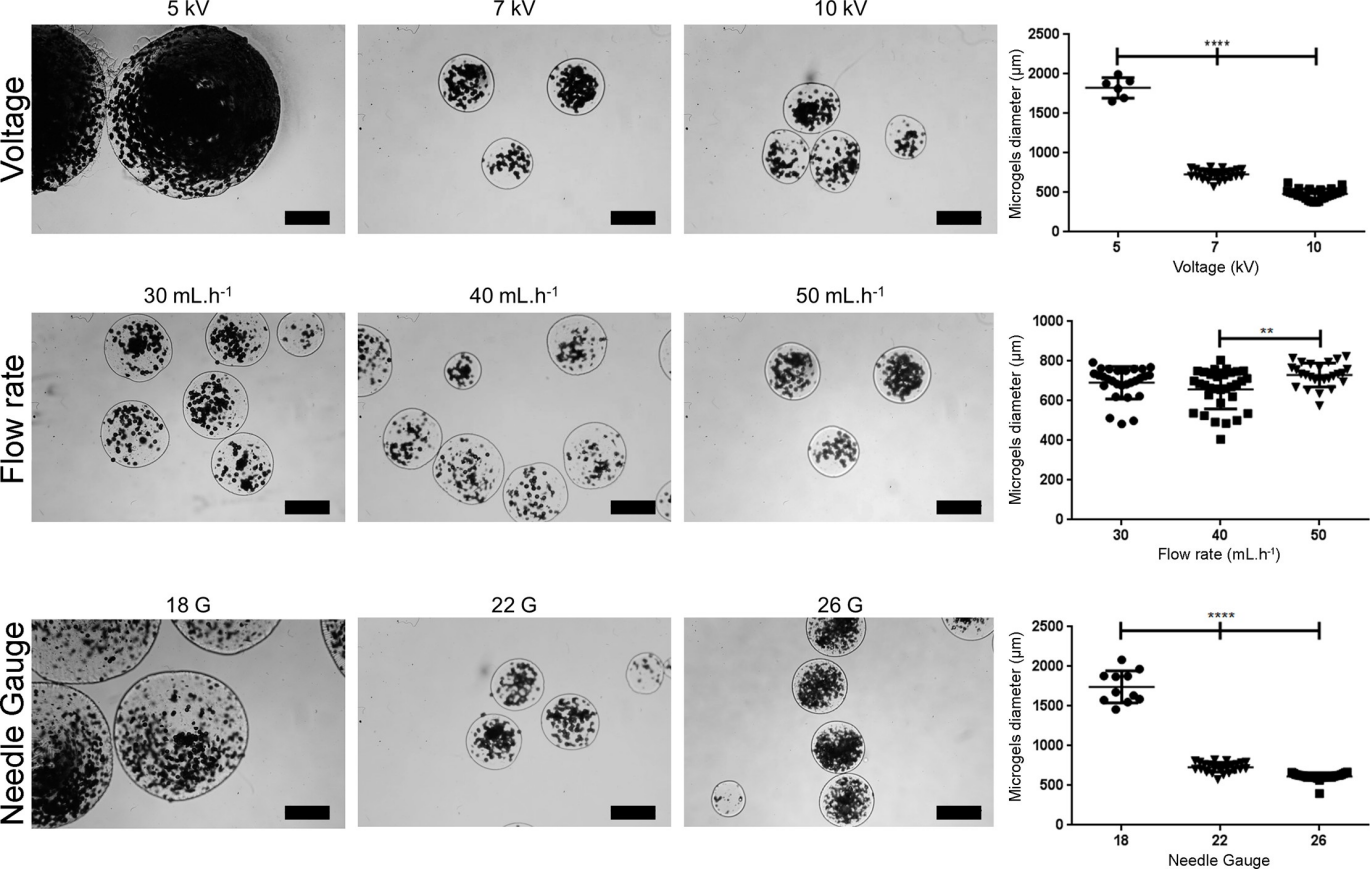
Influence of the voltage, flow rate, and needle Gauge (G) on the diameter of microgels produced by electrohydrodynamic atomization. The parameters 7 kV, 50 mL.h^-1^, 22 G, 8 cm, presence of microparticles, and 2.5% w/v of alginate were kept constant for each parameter evaluation. Diameter measurements were performed using Image J software. Significant differences are marked with *p<0.05, **p<0.01, ***p<0.001, and ****p<0.0001 (two-way ANOVA, GraphPad Prism v6).

Alginate (low viscosity sodium alginate from brown algae, Sigma-Aldrich) is dissolved in 0.15 M sodium chloride (NaCl) containing 25 mM MES hydrate at pH 6.7 (**see note 3**). Then, this solution is sterilized by 0.22 μm filtration (**see note 4**). Cells and microparticles are added to the alginate solution according to the desired densities. After low stirring, this solution is extruded by EHDA. The EHDA parameters, namely, distance between the needle and the collector, needle Gauge (G), flow rate, and voltage, are set as desired (Fig 2). Alginate microgels are collected using a microsized sieve after 20 min at RT in calcium chloride (0.1M, pH 6.7) with low stirring.

#### Multilayered membrane

The multilayered membrane is produced using the LbL technique. For that, three different polyelectrolytes are employed, namely poly(L-lysine) hydrobromide (PLL, Mw ~ 30,000–70,000, Sigma-Aldrich), water-soluble chitosan chloride salt (CHT, PROTASAN UP CL 213, Novamatrix), and ALG (low viscosity sodium alginate from brown algae, Sigma-Aldrich). PLL and ALG are used at pH 6.7 and CHT at pH 6.3–6.4 (**see note 5**). All the polyelectrolytes (0.3 mg.mL^-1^) are dissolved in 0.15 M NaCl buffered with 25 mM MES hydrate. The microgels are first immersed in PLL, and then subsequently in ALG, CHT, and ALG again for 10 min each, and interleaved by 3 min of NaCl to remove the non-absorbed macromolecules. This procedure is repeated three times in order to achieve a 12-layered membrane.

#### Core liquefaction

Alginate microgels with a 12-layered membrane are liquified using ethylenediaminetetraacetic acid (EDTA). EDTA will remove the calcium ions bound to the G blocks of alginate in the core of the microgel, inducing its liquefaction. Immediately after LbL production, microgels are immersed in EDTA (0.02M, pH 6.7) for 5 min at RT **(see note 6**). During this process, the multilayered membrane will maintain its nanostructured arrangement ensuring the structural integrity of the resulting liquefied microcapsules.

### Notes and tips

1. Be aware of the low melting temperature of PCL (around 60°C).
2. It is important to perform the protein surface modification step immediately after plasma treatment since the induced reactivity of PCL microparticles is very unstable, and thus PCL can rapidly recover it hydrophobicity. The hydrophobicity recover is faster if PCL microparticles are stored in a dry environment.
3. Alginate hydrogels disintegrate in alkaline media. Therefore, working at pH 6.7 is a manner to limit this disintegration without jeopardizing cell viability.
4. Be aware of volume losses after sterilization of alginate solutions by 0.22 μm filtration. The higher the alginate concentration, the higher the volume losses.
5. LbL is based on the adsorption of oppositely charged polyelectrolytes, so it is important to make sure that the chosen polymers keep their positive or negative charge. According to the dissociation constants (pKa) of ~ 10 for PLL, and 3.38 for mannuronic (M) and 3.65 for guluronic (G) acid alginate monomers, at pH 6.7 both PLL and carboxylate groups of alginate stay deprotonated. Since the pKa of chitosan is ~ 6.5, working pH should be 6.3–6.4 to assure a positive charge.
6. To dissolve EDTA it is required to increase the pH to 8 by adding sodium hydroxide. After complete dissolution, adjust the pH to 6.7 using hydrochloric acid.

## Influence of the EHDA process parameters on the size of the alginate microgels

**Fig 3:**
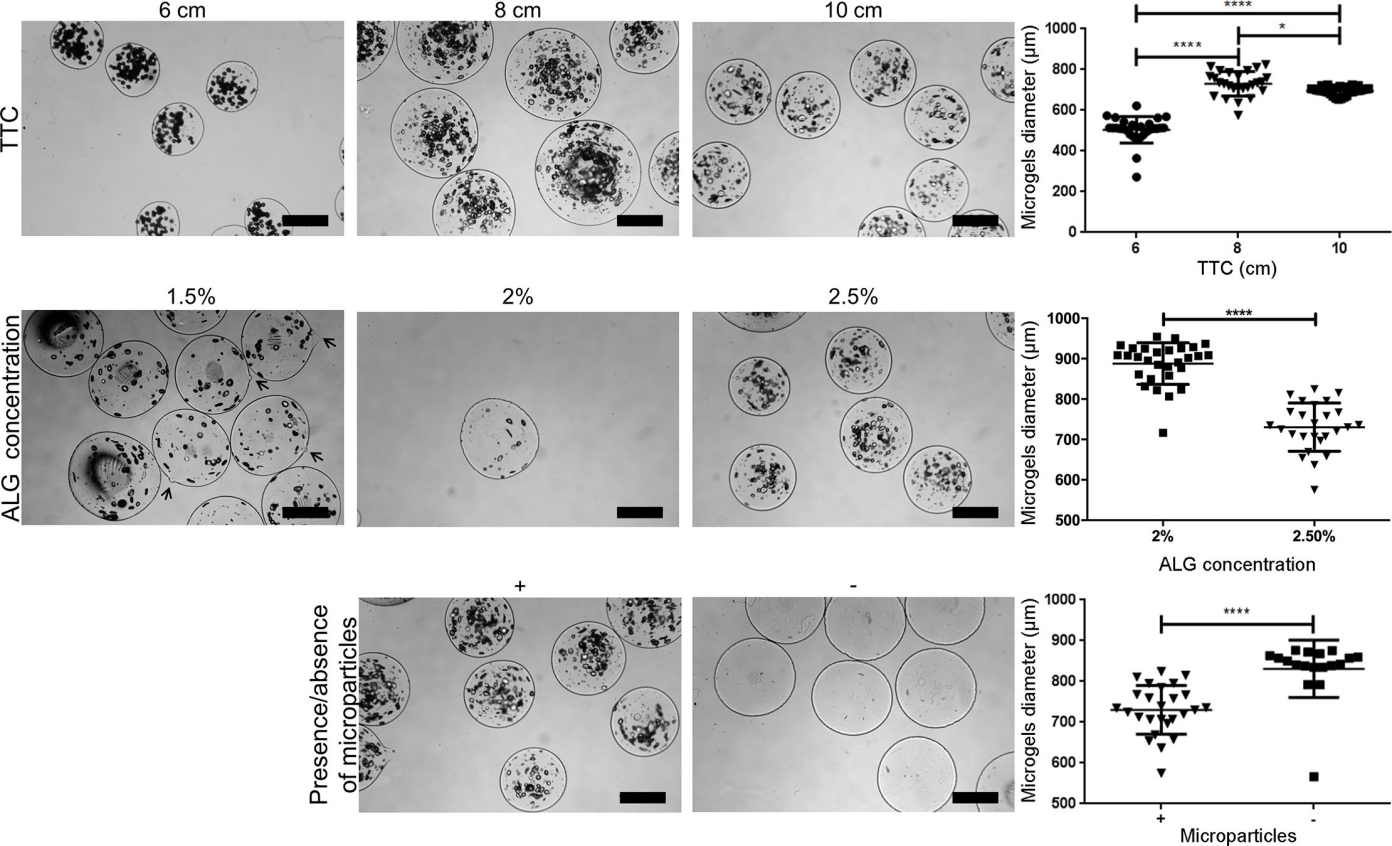
Influence of the distance between the tip of the needle to the collector (TTC), alginate concentration, and the presence/absence of microparticles on the diameter of alginate microgels produced by electrohydrodynamic atomization. The parameters 7 kV, 50 mL.h^-1^, 22 G, 8 cm, presence of microparticles, and 2.5% w/v of alginate were kept constant for each parameter evaluation. Diameter measurements were performed using Image J software. Significant differences are marked with **p<0.001 and ****p<0.0001 (two-way ANOVA, GraphPad Prism v6).

We tested the influence of different EHDA parameters on the diameter of the alginate microgels, which serve as templates for the production of the liquefied capsules discussed above. We varied the applied voltage (5, 7, and 10 kV), the flow rate (30, 40, and 50 mL.h^-1^), the needle diameter (18, 22, and 26 G), the distance between the needle and the collector (6, 8, and 10 cm), the presence/absence of microparticles, and the alginate concentration (1.5, 2, and 2.5% w/v). The parameters 7 kV, 50 mL.h^-1^, 22 G, 8 cm, presence of microparticles, and 2.5% w/v of alginate were kept constant for each parameter evaluation. Of note, the different parameters were all tested in the presence of a stable Tailor cone-jet mode. Results are summarized in Figs 2 and 3, and discussed individually in the following subsections.

**Fig 4:**
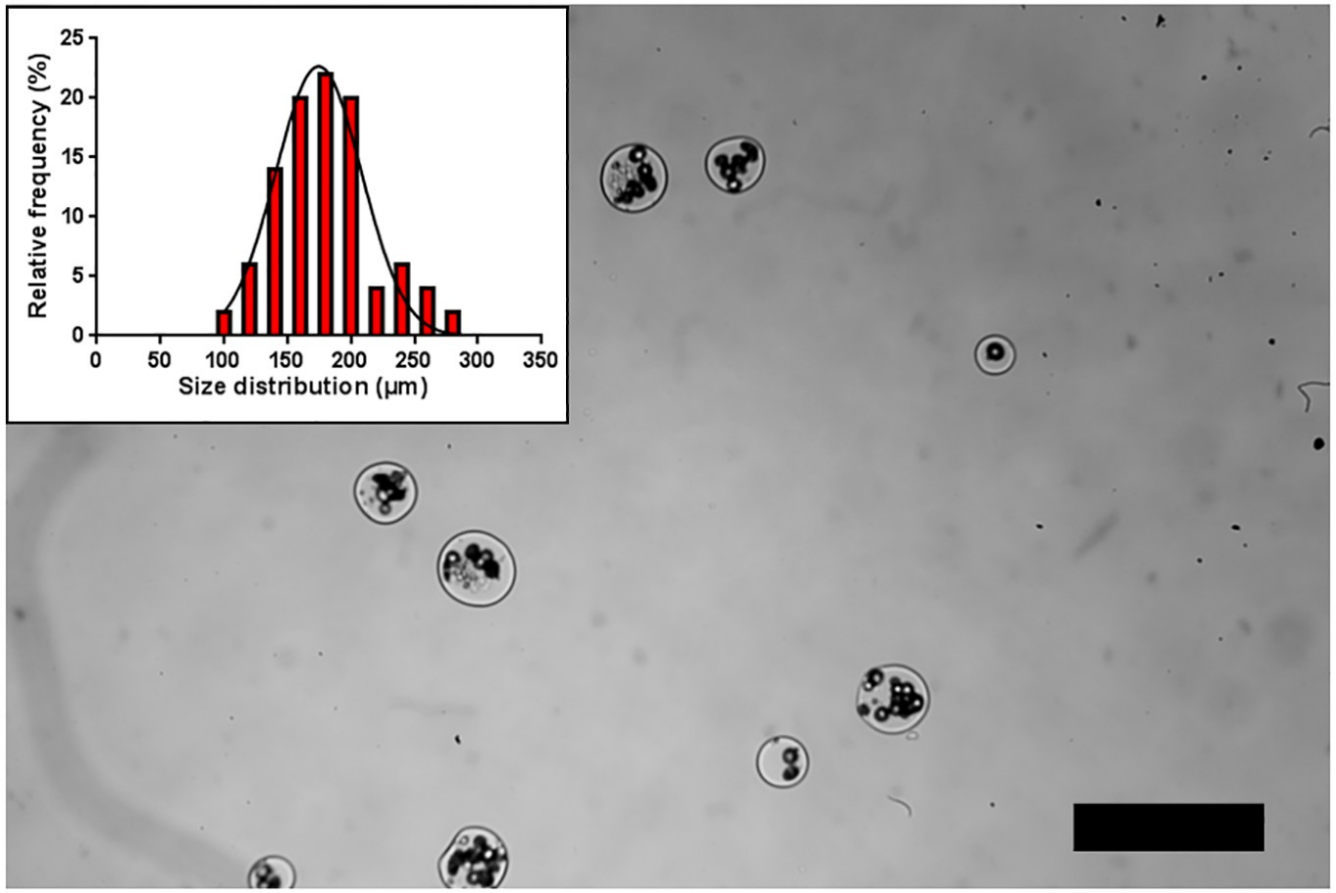
Production of alginate microgels with a diameter of 154 ± 31 μm. Microgels were obtained using 2.25% w/v of alginate, flow rate of 2 mL.h^-1^, 15 kV, TTC 7 cm, 26 G needle, and a diameter range of 20–40 μm for polycaprolactone microparticles.

The EHDA process is also known to be affected by other parameters that were not shown in the present study, such as the method of microgels collection, and the environmental conditions, namely temperature and relative humidity. Previously, we collected alginate microgels in a calcium chloride bath without stirring. However, the procedure originated non-spherical microgels inadequate to be used as templates for the production of liquefied and multilayered capsules. Therefore, low stirring was applied to collect all the above-referred conditions of alginate microgels. Regarding temperature and relative humidity, those parameters were not tested because they are not critical when aqueous solutions, such as alginate, are extruded. For the case of microparticles produced by EHDA requiring solidification by solvent evaporation, temperature and relative humidity are key parameters that should be monitored. In general, many different EHDA process parameters should be carefully considered for the successful production of alginate microgels. To demonstrate the feasibility of the EHDA process to produce high rates of microsized hydrogels, we also produced microgels with a diameter of 154 ± 31 μm (Fig 4). For the particular case of spherical hydrogels, reduction in diameter will enhance the transportation of oxygen and nutrients due to the fact that the surface-to-volume ratio will be increased. Moreover, it enables to extend the biomedical applicability of liquefied and multilayered capsules technology. Furthermore, it is known that small microgels present a better mechanical stability [17], which is a key aspect for liquefied systems. Moreover, microsized cell encapsulation matrixes present a larger surface to volume ratio, which enhances the access of oxygen and nutrients for the encapsulated cells compared to larger macrosized systems [30]. For this purpose, alginate microgels were prepared with a concentration of 2.25% w/v of alginate and electrosprayed with a flow rate of 2 mL.h^-1^, 15 kV, TTC 7 cm, and a 26 G needle. Additionally, prior to the encapsulation procedure, PCL microparticles were sieved to select a narrow diameter range of 20–40 μm, in order to avoid clogging during the EHDA process.

### Voltage

The applied voltage provides the driving force for the EHDA process, and thus is the parameter with greatest influence on the production of microgels. This parameter is more related with the type of jetting mode achieved, but since the evaluated parameters were all evaluated in the presence of a stable jetting mode, only its influence on the microgels diameter was evaluated. Overall, the results show that as the spray voltage increases the size of microbeads decreases (Fig 2). This difference is more accentuated by increasing the voltage from 5 to 7 kV, which resulted in a diameter decrease from 1826 ± 119 μm to 730 ± 59 μm.

### Flow rate

The flow rate is the second crucial EHDA parameter, since higher flow rates can easily destabilize the jetting mode and consequently led to a high polydispersity in microgels diameter. Since in the present study the different parameters were tested under a stable jetting mode, no significant differences were found when increasing the flow rate from 30 to 40 mL.h^-1^ (Fig 2). Increasing the flow rate to 50 mL.h^-1^ resulted in a slight diameter increase without increasing the polydispersity of the microgels diameter.

### Needle diameter

Needle diameter or needle G is also an important parameter that affects microgels diameter due to its influence on the diameter of the base of the Taylor cone. As demonstrated, 18 G needle led to macrogels with diameters above 1.5 mm, compared to the microsized hydrogels obtained with 22 G and 26 G (Fig 2).

### Tip to collector distance

The distance between the tip of the needle to the collector (TTC) affects the electrical field strength. Shorter distances increase the electrical potential, and led to the formation of smaller microgels. In accordance, Fig 3 shows that increasing the TTC from 6 cm to 8 cm led to a significant increase in the diameter from 504 ± 64 μm to 730 ± 59μm. On the other hand, at 10 cm a slight decrease was detected. This is due to the fact that the voltage was maintained constant at 7 kV and long TTC distances require higher applied voltage to compensate the reduced electrical field strength.

### Presence/Absence of microparticles

The presence or absence of microparticles also have an influence on the diameter of alginate microgels since it influences the solution conductivity. Fig 3 shows that microgels encapsulating microparticles presented a smaller diameter in comparison with empty microgels.

### Concentration of alginate

The concentration of alginate will affect the viscosity of the solution, and thus affect the diameter of the microgels. Higher viscosities led to smaller microgels as evidenced in Fig 3. For 1.5% w/v of alginate, microgels with a slight tear-like morphology were found (arrows of Fig 3), and thus their diameter was not measured. Increasing the concentration of alginate from 2% w/v to 2.5% w/v led to a significant diameter decrease from 888 ± 50 μm to 730 ± 59 μm.

## Conclusions and future perspectives

EHDA is a very versatile technology with many advantages for cell encapsulation, particularly in terms of the reproducibility of the processed microsize hydrogels. Given its simplicity and high yield when compared to other techniques, such as microfluidics, this method has shown to be a reliable technique to produce hydrogels in different size ranges and with controlled morphology and homogeneity. Therefore, we envision that its promising and increasing prominence in the TERM field will continue to flourish.

## Supporting information

S1 File GraphPad Prism v6 raw data for the production of the graphics presented in Figs 2–4. (ZIP)
